# Effects of Mavacamten on Cardiac Magnetic Resonance Features in Chinese Patients With Obstructive Hypertrophic Cardiomyopathy

**DOI:** 10.1016/j.jacasi.2025.05.015

**Published:** 2025-07-08

**Authors:** Zhuang Tian, Liwen Li, Xiaoyan Li, Qing Zhang, Daoquan Peng, Wei Ma, Ping Yang, Fang Wang, Xiang Cheng, Yiqun Fu, Jing Sun, Jian’an Wang, Shuyang Zhang

**Affiliations:** a Peking Union Medical College Hospital, Chinese Academy of Medical Sciences and Peking Union Medical College, Beijing, China; b Department of Cardiology, Guangdong Cardiovascular Institute, Guangdong Provincial People’s Hospital, Guangdong Academy of Medical Sciences, Southern Medical University, Guangzhou, China; c Renmin Hospital of Wuhan University, Hubei General Hospital, Wuhan, China; d West China Hospital, Sichuan University, Chengdu, China; e The Second Xiangya Hospital of Central South University, Changsha, China; f Peking University First Hospital, Beijing, China; g China-Japan Union Hospital of Jilin University, Changchun, China; h Department of Cardiology, Beijing Hospital, National Center of Gerontology; Institute of Geriatric Medicine, Chinese Academy of Medical Sciences, Beijing, China; i Union Hospital, Tongji Medical College, Huazhong University of Science and Technology, Wuhan, China; j Shanghai LianBio Development Co. Ltd, Shanghai, China; k Bristol Myers Squibb, Shanghai, China; l The Second Affiliated Hospital of Zhejiang University School of Medicine, Hangzhou, China

**Keywords:** cardiac magnetic resonance, mavacamten, obstructive hypertrophic cardiomyopathy

## Abstract

**Background:**

Mavacamten, a first-in-class cardiac myosin inhibitor and the only approved cardiac myosin inhibitor worldwide, improved clinical symptoms and health status in patients with symptomatic obstructive hypertrophic cardiomyopathy (HCM) in phase 3 EXPLORER-HCM (Clinical Study to Evaluate Mavacamten [MYK-461] in Adults With Symptomatic Obstructive Hypertrophic Cardiomyopathy; NCT03470545) and EXPLORER-CN (A Study to Evaluate the Efficacy and Safety of Mavacamten in Chinese Adults With Symptomatic Obstructive HCM; NCT05174416).

**Objectives:**

The purpose of this work was to study the effect of mavacamten on cardiac structure and function by cardiac magnetic resonance (CMR) imaging in Chinese participants in EXPLORER-CN.

**Methods:**

Eligible patients with obstructive HCM underwent CMR imaging at screening and week 30. Change from baseline to week 30 in left ventricular (LV) mass index was analyzed as a prespecified secondary outcome. Prespecified exploratory outcomes included changes in cellular hypertrophy, cardiac structure and function, and myocardial fibrosis by CMR.

**Results:**

Among 81 patients randomized, 58 patients (mean age 51.2 years, 74.1% men) with CMR data available were analyzed (mavacamten, n = 39; placebo, n = 19). After 30 weeks, greater reductions from baseline were observed (mean between-group difference) with mavacamten vs placebo in LV mass index (−30.8 g/m^2^ [95% CI: −41.5 to −20.1 g/m^2^]), maximal LV wall thickness (−3.5 mm [95% CI: −4.7 to −2.4 mm]), and maximal left atrial volume index (−18.3 mL/m^2^ [95% CI: −26.7 to −9.8 mL/m^2^]); all nominal *P <* 0.001. Reduction from baseline to week 30 in global mass of late gadolinium enhancement by 6 SDs was also observed with mavacamten vs placebo (mean between-group difference, −2.0 g [95% CI: −11.9 to 8.0 g]; nominal *P =* 0.007).

**Conclusions:**

At 30 weeks, improvements were observed in measures of cardiac structure and function, with reductions in indicators of myocardial fibrosis, in the mavacamten vs the placebo group. (A Study to Evaluate the Efficacy and Safety of Mavacamten in Chinese Adults With Symptomatic Obstructive HCM; NCT05174416)

Hypertrophic cardiomyopathy (HCM) is a myocardial disorder characterized by left ventricular (LV) hypertrophy, impaired LV dilation, diastolic dysfunction, and hyperdynamic contraction, resulting in worsening symptoms and quality of life.^1^^,^^2^ Ventricular hypertrophy is the principal feature^1^ and forms the cornerstone of clinical diagnosis of HCM, typically detected via cardiac imaging.^3^

Noninvasive imaging modalities, including cardiac magnetic resonance (CMR), are fundamental to the diagnosis and monitoring of patients with cardiomyopathies. CMR imaging offers high spatial resolution, an expansive field of view, exceptional tissue characterization capabilities, and accurate assessments of cardiac chamber dynamics, which are especially relevant for HCM.^4^^,^^5^ CMR also provides prognostic value for sudden cardiac death in terms of myocardial fibrosis.^6^^,^^7^ Therefore, CMR is increasingly recognized as one of the multimodal imaging tools crucial for characterization of cardiac morphology and function in the diagnostic pathway of cardiomyopathy in the latest guidelines for cardiomyopathy.^3^

Mavacamten, a first-in-class cardiac myosin inhibitor (CMI), can reduce myocardial contractility and ventricular stiffness by reducing the formation and numbers of actin–myosin cross bridges, which underlie the pathogenesis of obstructive HCM. Mavacamten is the only CMI to receive regulatory approval for the treatment of obstructive HCM. Currently, mavacamten is guideline-recommended for patients with NYHA functional class II to III obstructive HCM who are intolerant or remain symptomatic despite optimal medical therapy.^3^ The efficacy and safety of mavacamten have been demonstrated in the global phase 3 EXPLORER-HCM (Clinical Study to Evaluate Mavacamten [MYK-461] in Adults With Symptomatic Obstructive Hypertrophic Cardiomyopathy) study, which showed significant improvements in LV outflow tract gradients, exercise capacity, and health status after 30 weeks in adults with obstructive HCM.^8^ Mavacamten also resulted in favorable changes in cardiac structure in the CMR substudy of EXPLORER-HCM.^9^^,^^10^ A phase 3 study conducted solely in China, EXPLORER-CN (A Study to Evaluate the Efficacy and Safety of Mavacamten in Chinese Adults With Symptomatic Obstructive HCM), further confirms that the clinical benefits of mavacamten also extend to the Chinese population.^11^

Here, we conducted a secondary analysis of EXPLORER-CN to evaluate the effect of mavacamten on key CMR features in Chinese patients with obstructive HCM and how these changes relate to its observed benefits in cardiac biomarkers and clinical symptoms.

## Methods

### Study design and patients

EXPLORER-CN (NCT05174416) was a double-blind, placebo-controlled, multicenter, randomized, phase 3 trial in patients with symptomatic obstructive HCM, conducted at 12 sites in China. Details of the study design and primary efficacy and safety results were reported previously.^11^^,^^12^ Here, we report CMR results for the subgroup of patients with data available. This study was conducted according to the principles of the Declaration of Helsinki and Guideline for Good Clinical Practice. The protocol was approved by Institutional Review Boards/ethics committees at all sites. All patients provided written informed consent before participation.

Details of the patient population were published previously.^11^ Additional exclusion criteria for CMR assessments were use of implantable cardioverter-defibrillator/pacemaker, conditions contraindicated against CMR, atrial fibrillation at screening, and contraindications/allergy to gadolinium-based contrast agents. A full list of eligibility criteria is provided in Supplemental Table 1.

### Procedures and assessments

CMR imaging was conducted at screening and week 30 (or end of treatment), according to a detailed protocol, by certified CMR technologists at each study site and assessed by specialist CMR physician experts at the core laboratory. CMR imaging was performed using 1.5- or 3-T scanners (Siemens, Philips, or GE Healthcare) with cardiac or torso phased-array surface coils. The CMR sequences consisted of the following: 1) scout imaging series including 3-plane scout (3 slices/plane)—vertical long-axis scout, horizontal long-axis scout, and midlevel short-axis scout; 2) left and right ventricular structure and function series, involving 4-chamber long-axis cine followed by short-axis cine covering both left atrium and ventricle; 3) native (precontrast) T_1_ mapping with Modified Look-Locker Inversion Recovery acquired at 3 equally spaced short-axis cuts at basal, mid, and distal levels matching segments 1 to 16 of the American Heart Association 17-segment model; 4) gadolinium-based contrast injection at 0.15 mmol/kg followed immediately by short-axis scout imaging at the midlevel of the left ventricle; 5) high temporal resolution 4-, 3-, and 2-chamber long-axis cines; 6) repeat T_1_ mapping at 5, 10, and 25 minutes after contrast injection; and 7) late gadolinium enhancement (LGE) imaging matching short-axis stack cine covering the entire LV myocardium at 15 minutes postcontrast.

### Outcome

The change in left ventricular mass index (LVMI) from baseline to week 30, as assessed by CMR imaging, was evaluated as a secondary efficacy endpoint. Prespecified exploratory endpoints based on CMR assessments included changes from baseline (CFB) to week 30 in the following parameters: myocardial fibrosis, cellular hypertrophy, and cardiac structure and function. Cardiac structure was measured as LV mass, LVMI, LV wall thickness (maximum and minimum), and left atrial volume index (LAVI) (maximum and minimum). Measures of cardiac output and function included LV ejection fraction, LV end-diastolic and -systolic volumes and corresponding indexes, LV stroke volume and corresponding index, contractile fraction, myocardial contraction fraction, and cardiac output and cardiac output index. Myocardial fibrosis was evaluated based on global mass of LGE by 6 SDs (LGE 6SD) as the threshold and extracellular volume fraction.

### Statistical analyses

Only patients who met the CMR eligibility criteria, with CMR data available at both baseline and postbaseline, were included in the CMR set. Values of CMR parameters were summarized using descriptive statistics. For analyses comparing the changes in CMR parameters from baseline to week 30, distributions of CFB to week 30 between groups were compared using Wilcoxon rank-sum test, and 95% CIs for the between-group differences were calculated based on normal approximation. Descriptive analyses and scatter plots were used to describe the relationship between the change in CMR parameters and cardiac biomarkers.

Simple linear regression was fitted by treatment group on the change in cardiac biomarkers (N-terminal pro–B-type natriuretic peptide [NT-proBNP] and high-sensitivity cardiac troponin I) with the change in LVMI as the explanatory variable. The fitted lines were overlaid with scatter plots.

Statistical tests were conducted as 1-sided tests; all *P* values denote nominal value and statistical tests were descriptive without multiplicity adjustment. Missing data were not imputed. SAS version 9.4 was used for statistical analyses.

## Results

### Patient characteristics

Of the 81 patients with symptomatic obstructive HCM who were randomized in EXPLORER-CN (2:1 mavacamten to placebo), 58 patients (mean age 51.2 years, 74.1% [43 of 58] men, mean body mass index 25.4 kg/m^2^, 75.9% [44 of 58] NYHA functional class II at baseline) with CMR data available were included for CMR analysis (mavacamten, n = 39; placebo, n = 19). Data cutoff was March 6, 2023. Baseline demographics and key CMR measures are shown in Table 1. Baseline demographic characteristics were generally balanced between groups, except for a larger proportion of men, poor CYP2C19 metabolizers, and patients with NYHA functional class II status in the mavacamten group than the placebo group (Table 1). For key CMR parameters, most features were broadly similar at baseline between the treatment groups, although LVMI and LAVI appeared to be lower in the mavacamten group than the placebo group. CMR values were typical of HCM, with increased LVMI, maximal LV wall thickness, and left atrial enlargement indicated by LAVI (Table 1). LGE 6SD values were indicative of myocardial scarring. Both groups showed a similar mild elevation of LV ejection fraction at baseline and no patients had LV ejection fraction <50%. Most patients were on background beta-blocker (89.7% [52 of 58]) or calcium channel blocker (6.9% [4 of 58]) therapy. All patients in the CMR set completed the 30-week, double-blind, placebo-controlled treatment period.

**Table 1:** Patient Baseline Demographics and CMR Features

	Mavacamten Group (n = 39)	Placebo Group (n = 19)
Age, y	51.0 ± 12.2	51.6 ± 10.9
Age group, y		
≤49	17 (43.6)	9 (47.4)
50–64	18 (46.2)	8 (42.1)
≥65	4 (10.3)	2 (10.5)
Sex		
Male	31 (79.5)	12 (63.2)
Female	8 (20.5)	7 (36.8)
Vital signs		
Body mass index, kg/m^2^	25.1 ± 3.2	25.8 ± 3.4
Heart rate, beats/min	64.4 ± 7.8	65.9 ± 7.8
Systolic blood pressure, mm Hg	117.9 ± 10.9	110.4 ± 13.5
Diastolic blood pressure, mm Hg	74.1 ± 9.6	70.3 ± 9.7
NYHA functional class		
II	31 (79.5)	13 (68.4)
III	8 (20.5)	6 (31.6)
CYP2C19 phenotype and genotype		
Normal	17 (43.6)	7 (36.8)
Intermediate	16 (41.0)	12 (63.2)
Poor	6 (15.4)	0
Background therapy for HCM		
Beta-blocker	36 (92.3)	16 (84.2)
Calcium channel blocker	2 (5.1)	2 (10.5)
Others	1 (2.6)	1 (5.3)
Key CMR parameters		
LVMI, g/m^2^	98.6 ± 45.0	108.5 ± 54.8
LV maximal wall thickness, mm	16.7 ± 5.5	17.6 ± 5.0
Maximum LAVI, mL/m^2^	59.6 ± 19.3	66.0 ± 23.8
Minimum LAVI, mL/m^2^	34.8 ± 14.9	40.5 ± 18.6
LVEF, %	72.1 ± 6.8	70.8 ± 10.5
Myocardial contraction fraction, %	75.2 ± 27.5	71.5 ± 26.3
Contractile function, %	71.6 ± 26.2	68.1 ± 25.0
Global mass of LGE 6SD, g^a^	8.9 ± 11.3	9.3 ± 17.3
5-min global ECVF, %^b^	27.7 ± 3.6	29.5 ± 4.5
10-min global ECVF, %^c^	28.0 ± 3.0	29.1 ± 3.9
25-min global ECVF, %^d^	28.5 ± 3.2	30.9 ± 5.4

Values are mean ± SD or n (%).

CMR = cardiac magnetic resonance; ECVF = extracellular volume fraction; HCM = hypertrophic cardiomyopathy; LAVI = left atrial volume index; LGE 6SD = late gadolinium enhancement by 6 SDs; LV = left ventricular; LVEF = left ventricular ejection fraction; LVMI = left ventricular mass index.

a 36 and 15 patients for mavacamten and placebo, respectively.

b 35 and 17 patients for mavacamten and placebo, respectively.

c 36 and 18 patients for mavacamten and placebo, respectively.

d 38 and 19 patients for mavacamten and placebo, respectively.

### Structural changes

**Figure 1:**
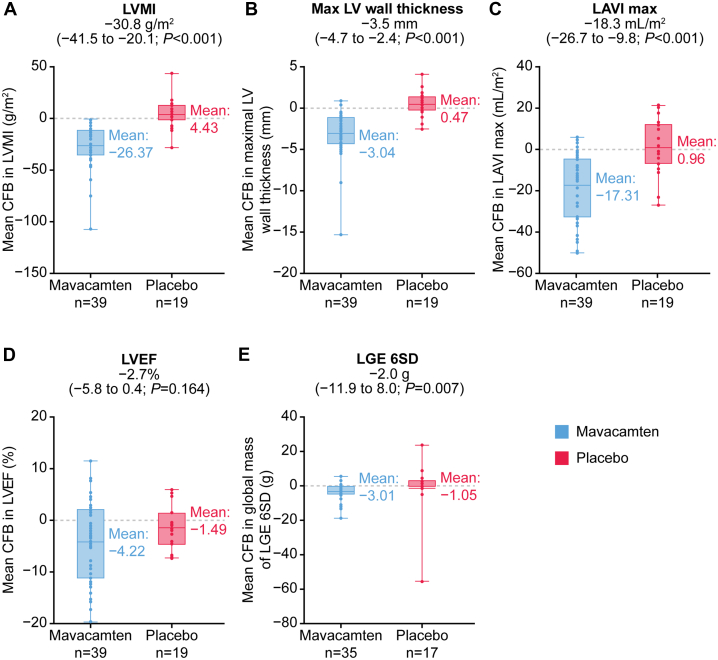
CFB in CMR Parameters of Cardiac Structure, Function, and Fibrosis With Mavacamten vs Placebo Improvements with mavacamten vs placebo in mean change from baseline (CFB) (95% CI) at week 30 for left ventricular mass index (LVMI) (A), maximal left ventricular (LV) wall thickness (B), left atrial volume index (LAVI) Max (C), left ventricular ejection fraction (LVEF) (D), and global mass of late gadolinium enhancement by 6 SDs (LGE 6SD) (E). CFB = change from baseline; CMR = cardiac magnetic resonance; LAVI = left atrial volume index; LGE 6SD = late gadolinium enhancement by 6 standard deviations; LV = left ventricular; LVEF = left ventricular ejection fraction; LVMI = left ventricular mass index.

The mavacamten group showed a reduction in mean ± SD LVMI, from 98.6 ± 45.0 g/m^2^ at baseline to 72.2 ± 31.0 g/m^2^ at week 30, compared with an increase from 108.5 ± 54.8 g/m^2^ to 112.9 ± 59.5 g/m^2^ in the placebo group (Figure 1A). Mean difference between groups was −30.8 g/m^2^ (95% CI: −41.5 to −20.1 g/m^2^; *P <* 0.001) (Table 2), associated with a mean ± SD decrease in LV mass by 46.3 ± 35.1 g in the mavacamten group at week 30 from baseline vs a slight increase of 6.3 ± 22.3 g in the placebo group (mean difference between groups, −52.6 g [95% CI: −67.9 to −37.4 g]; *P <* 0.001). A similar trend was observed for global maximal LV wall thickness with mavacamten vs placebo (mean CFB at week 30, −3.0 vs 0.5 mm; mean difference between groups, −3.5 mm [95% CI: −4.7 to −2.4 mm]; *P <* 0.001) (Figure 1B). Both maximum and minimum LAVI (mean CFB at week 30, −17.3 and −10.4 mL/m^2^, respectively) were reduced from baseline in the mavacamten group compared with no changes in the placebo group (1.0 and −0.1 mL/m^2^, respectively). Mean differences between groups were −18.3 mL/m^2^ (95% CI: −26.7 to −9.8) for maximum LAVI (Figure 1C) and −10.3 mL/m^2^ (95% CI: −15.9 to −4.7 mL/m^2^) for minimum LAVI (*P <* 0.001 for both). Median (IQR) for change from baseline at week 30 is consistent for all the CMR measures above, as presented in Supplemental Table 2.

**Table 2:** CFB in CMR Parameters at Week 30

Mean Change From Baseline at Week 30	Mavacamten (n = 39)	Placebo (n = 19)	Mean Difference (95% CI)	Nominal *P* Value^a^
Cardiac structure				
LVMI, g/m^2^	−26.4	4.4	−30.8 (−41.5 to −20.1)	<0.001
LV mass, g	−46.3	6.3	−52.6 (−67.9 to −37.4)	<0.001
Maximal LV wall thickness, mm	−3.0	0.5	−3.5 (−4.7 to −2.4)	<0.001
Maximum LAVI, mL/m^2^	−17.3	1.0	−18.3 (−26.7 to −9.8)	<0.001
Minimum LAVI, mL/m^2^	−10.4	−0.1	−10.3 (−15.9 to −4.7)	<0.001
Cardiac output and function				
LVEF, %	−4.2	−1.5	−2.7 (−5.8 to 0.4)	0.164
Myocardial contraction fraction, %	10.1	−2.5	12.6 (3.2-22.0)	0.002
Contractile fraction, %	9.6	−2.4	12.0 (3.0-21.0)	0.002
LVEDV, mL	−14.3	0.9	−15.2 (−24.5 to −5.9)	0.002
LVEDV index, mL/m^2^	−8.1	1.2	−9.3 (−14.7 to −4.0)	<0.001
LVESV, mL	2.0	3.0	−1.0 (−6.0 to 4.0)	0.728
LVESV index, mL/m^2^	1.1	1.9	−0.9 (−4.2 to 2.5)	0.596
LVSV, mL	−16.3	−2.1	−14.2 (−22.9 to −5.4)	0.003
LVSV index, mL/m^2^	−9.1	−0.7	−8.4 (−13.5 to −3.4)	0.002
CO, mL/min	−857.3	−80.0	−777.3 (−1,657.6 to 103.0)	0.064
COI, mL/min/m^2^	−488.2	−2.1	−486.1 (−975.9 to 3.8)	0.051
Myocardial fibrosis				
Global mass of LGE 6SD, g^b^	−3.0	−1.1	−2.0 (−11.9 to 8.0)	0.007
5-min global ECVF, %^c^	1.5	0.2	1.3 (−0.7 to 3.4)	0.201
10-min global ECVF, %^d^	1.2	0.5	0.7 (−1.0 to 2.4)	0.401
25-min global ECVF, %^e^	0.9	−0.7	1.6 (−0.2 to 3.5)	0.144

CFB = changes from baseline; CO = cardiac output; COI = cardiac output index; LVEDV = left ventricular end-diastolic volume; LVESV = left ventricular end-systolic volume; LVSV = left ventricular stroke volume; other abbreviations as in Table 1.

a By Wilcoxon rank-sum test. *P* values denote nominal values.

b 36 patients on mavacamten and 15 on placebo at baseline, and 32 and 14 patients at week 30, respectively.

c 35 patients on mavacamten and 17 on placebo at baseline, and 34 and 16 patients at week 30, respectively.

d 36 patients on mavacamten and 18 on placebo at baseline, and 34 and 17 patients at week 30, respectively.

e 38 patients on mavacamten and 19 on placebo at baseline, and 37 and 16 patients at week 30, respectively.

### Cardiac output and function changes

At week 30, the mavacamten group had a greater reduction in LV stroke volume, an indicator of LV systolic function (mean CFB, −16.3 mL vs −2.1 mL; mean difference between groups, −14.2 mL [95% CI: −22.9 to −5.4 mL]; *P =* 0.003) and the LV stroke volume index (mean CFB, −9.1 mL/m^2^ vs −0.7 mL/m^2^; mean difference between groups, −8.4 mL/m^2^ [95% CI: −13.5 to −3.4 mL/m^2^]; *P =* 0.002) compared with the placebo group. LV end-diastolic volume (mean CFB, −14.3 mL vs 0.9 mL; mean difference between groups, −15.2 mL [95% CI: −24.5 to −5.9 mL]; *P =* 0.002) and the corresponding index (mean CFB, −8.1 mL/m^2^ vs 1.2 mL/m^2^; mean difference between groups, −9.3 mL/m^2^ [95% CI: −14.7 to −4.0 mL/m^2^]; *P <* 0.001) were also reduced in the mavacamten group, while no changes were seen in the placebo group. Similarly, myocardial contraction fraction ([LV stroke volume/LV myocardial volume] × 100; LV myocardial volume = LV mass/[1.05 g/mL]) (mean CFB, 10.1% vs −2.5%; mean difference between groups, 12.6% [95% CI: 3.2%-22.0%]) was improved in favor of mavacamten at week 30 *(P =* 0.002 for both). Although reductions in cardiac output (mean difference between groups, −777.3 mL/min [95% CI: −1,657.6 to 103.0 mL/min]) and cardiac output index (mean difference between groups, −486.1 mL/min/m^2^ [95% CI: −975.9 to 3.8 mL/min]) were observed in the mavacamten group vs the placebo group, the differences between groups were not significant.

In both the mavacamten and placebo groups, LV ejection fraction decreased slightly from baseline (mean, 72.1% vs 70.8%, respectively) to week 30 (67.9% vs 69.3%, respectively), with no obvious difference between groups (mean CFB at week 30, −4.2% vs −1.5%; mean difference between groups, −2.7% [95% CI: −5.8% to 0.4%]; *P =* 0.164) (Figure 1D). No LV ejection fraction fell to <50% by CMR during the study. Median (IQR) for change from baseline at week 30 is consistent for all the CMR measures above, as presented in Supplemental Table 2.

### Myocardial fibrosis

Greater reduction in global mass of LGE 6SD from baseline to week 30 was observed in the mavacamten group compared with the placebo group (mean CFB, −3.0 g vs −1.1 g; mean difference between groups, −2.0 g [95% CI: −11.9 to 8.0 g]; *P =* 0.007) (Figure 1E). The change in global mass of LGE 6SD appeared to be independent of the change in LV mass (Pearson correlation coefficient, 0.2682) (Supplemental Figure 1). Changes in another biomarker of myocardial fibrosis, extracellular volume fraction, were similar in both treatment groups regardless of whether this was measured at 5, 10, or 25 minutes postcontrast administration. Median (IQR) for change from baseline at week 30 is consistent for all the CMR measures above, as presented in Supplemental Table 2.

### Correlation between CMR parameters and other pharmacodynamic parameters

**Figure 2:**
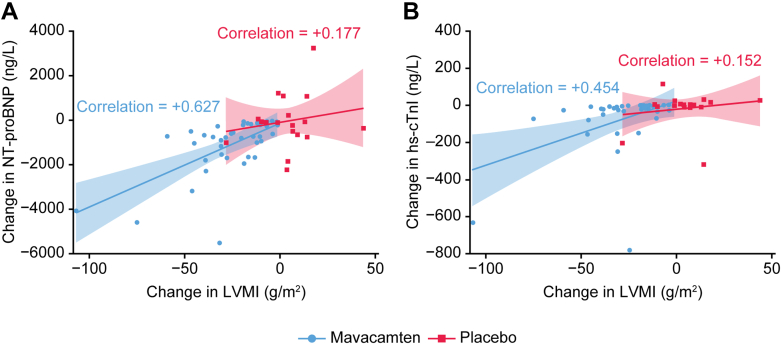
Changes in LVMI vs Cardiac Biomarkers Scatter plots of correlations between change in left ventricular mass index (LVMI) and changes in N-terminal pro–B-type natriuretic peptide (NT-proBNP) (A) and high-sensitivity cardiac troponin I (hs-cTnI) (B) levels. Shaded areas represent 95% CI of the regression line for the mavacamten group (blue) and the placebo group (red). Correlation shows Pearson correlation coefficient.

Scatter plots seem to indicate a modest correlation between LVMI and cardiac biomarkers of LV wall stress or myocardial injury, such as NT-proBNP (Pearson correlation coefficient, +0.627) and high-sensitivity cardiac troponin I, in the mavacamten group (Pearson correlation coefficient, +0.454) (Figure 2). Box plots appear to indicate that patients who achieved NYHA functional class improvement experienced greater reduction in LVMI, maximal LV wall thickness, LV end-diastolic volume index, maximum LAVI, and global mass of LGE 6SD compared with those who had no NYHA functional class improvement (Supplemental Figure 2).

## Discussion

To our knowledge, this subanalysis of EXPLORER-CN represents the first CMR study in Chinese patients receiving CMI and is the largest assessment of CMR parameters to date in patients with obstructive HCM receiving pharmacological therapy in a randomized trial. Our results indicate that the mavacamten group had a larger improvement in measures related to cardiac structure, volume, and function, compared with the placebo group (Central Illustration). Also, myocardial fibrosis (indicated by global mass of LGE 6SD) appeared to be reduced in the mavacamten vs the placebo group, although this requires validation in further studies. Improvements in these key CMR parameters, which are prognostic factors associated with adverse long-term outcomes in HCM, including heart failure, atrial fibrillation, and death,^13^, ^14^, ^15^ suggest that patients with obstructive HCM treated with mavacamten experienced a favorable cardiac remodeling. Importantly, these changes were observed as soon as after 30 weeks of treatment, indicating that mavacamten is the first pharmacological intervention to show such benefit in obstructive HCM. Importantly, our study is the first to demonstrate that the favorable treatment effects of mavacamten on CMR parameters extend to diverse groups of patients with obstructive HCM, including the Chinese population.

**Central Illustration:**
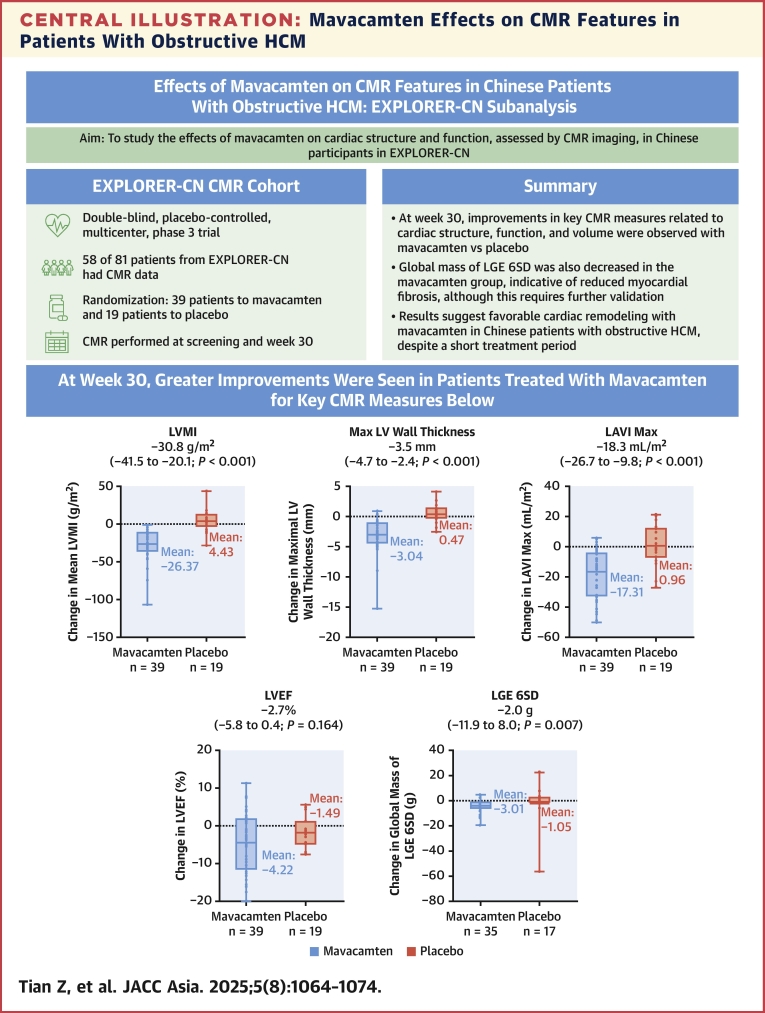
Mavacamten Effects on CMR Features in Patients With Obstructive HCM Chinese participants with cardiac magnetic resonance (CMR) data available from EXPLORER-CN were included to analyze the effects of mavacamten vs placebo on cardiac structure and function. HCM = hypertrophic cardiomyopathy; LAVI = left atrial volume index; LGE 6SD = late gadolinium enhancement by 6 SDs; LV = left ventricular; LVEF = left ventricular ejection fraction; LVMI = left ventricular mass index.

The CMR findings in this analysis were largely consistent with those previously reported for CMIs.^8^^,^^16^ In our study, both LVMI and maximal LV wall thickness assessed by CMR were mildly elevated at baseline and substantially reduced after 30 weeks of treatment with mavacamten, in line with observations from the global EXPLORER-HCM CMR substudy.^8^ A high LV wall thickness has been associated with increased risk of systolic dysfunction and sudden cardiac death,^17^^,^^18^ while LVMI is known to provide important prognostic value with regard to the risk of disease progression and adverse outcomes in patients with HCM.^13^ Similarly, LAVI is also a prognostic factor for disease progression and major adverse cardiac and cerebrovascular events in patients with HCM,^15^ including atrial fibrillation and heart failure.^14^^,^^19^ In our study, maximum LAVI was reduced by 18.3 mL/m^2^ in the mavacamten group, consistent with a reduction of 10.3 mL/m^2^ seen in EXPLORER-HCM. Improvements in maximum LAVI were most likely multifactorial, caused by improvements in LVMI and diastolic function, reductions in mitral regurgitation observed on echocardiography, and a possible direct beneficial effect on atrial myocardium. Notably, decreases in these parameters were accompanied by a reduction in LV outflow tract gradient reported in the primary analysis.^11^ Despite the substantial reductions in these LV parameters, none fell below the normal range. As ventricular hypertrophy is a contributing feature of the dynamic LV outflow tract gradient characteristics of obstructive HCM,^1^ the ability of mavacamten to reduce LV hypertrophy markers mentioned above—all of which are predictors of poor outcomes^1^—holds important implications for patients with HCM.

Progression of myocardial fibrosis in HCM occurs gradually over time. Intriguingly, global mass of LGE 6SD appeared to be reduced in the mavacamten group vs the placebo group (difference between groups, −2.0 g, nominal *P =* 0.007), despite a treatment period of just 30 weeks in our study. In the global EXPLORER-HCM CMR study, there were no notable within- or between-group changes in global mass of LGE 6SD at week 30 (difference between groups, 0.9 g; *P =* 0.89).^9^ It is also worth noting that there was minimal fibrosis present at baseline in EXPLORER-HCM. By contrast, the extent of fibrosis at baseline appears to be greater in the present EXPLORER-CN study, at a mean LGE mass of 8.9 and 9.3 g in the mavacamten and placebo groups, respectively. However, it should be noted that signal intensity for conventional LGE imaging sequences is typically expressed on an arbitrary scale that varies from one study to another, thus rendering direct signal quantification using LGE unsuitable for cross-sectional comparisons.^20^ Considering that cross-trial comparison is restricted by the inherent limitations of LGE, studies with larger populations and longer follow-ups are needed to further validate the long-term effects of CMIs on myocardial fibrosis. In HCM, the presence of extensive LGE is believed to indicate myocardial fibrosis or replacement scarring, which compromises LV function and contributes to the development of ventricular tachyarrhythmias.^21^^,^^22^ Extensive myocardial fibrosis has been linked to a greater risk for malignant ventricular arrhythmias, heart failure, and sudden death.^23^^,^^24^ In a gadolinium-enhanced CMR imaging study of patients with heart failure on beta-blocker therapy, myocardial scarring (indicated by volume of dysfunctional but viable myocardium) at baseline was inversely correlated to contractile improvement.^25^ Although the study was conducted in patients with heart failure rather than obstructive HCM, this indicates that further characterization of the morphology patterns of fibrosis may reveal further insights into the correlation between changes in myocardial fibrosis and the mechanism of action of mavacamten in obstructive HCM.

Favorable changes in structural and functional CMR parameters with mavacamten support the hypothesis that mavacamten addresses the underlying causal biology and improves negative cardiac remodeling associated with HCM, which is the basis for the well-documented clinical benefit. Although LV ejection fraction was reduced from baseline in both the mavacamten and placebo groups, no patients had LV ejection fraction <50% during the study. The slight decrease in LV ejection fraction was likely caused by a decrease in contractility, consistent with the mechanism of action of mavacamten.^26^ The reductions in LVMI and LV wall thickness, which are concordant with improved LV outflow tract gradient in the primary analysis, suggest that alleviation of LV outflow tract obstruction may contribute to a decrease in LV hypertrophy together with the direct effect of mavacamten in suppressing the formation of actin–myosin cross bridges. Mavacamten has also been shown to alleviate the hypercontractile state at several structural levels of sarcomere organization, which is highly desirable for HCM as hypercontractility contributes to cardiac hypertrophy, and thus, HCM disease progression.^27^ The resulting LV wall stress and increased workload can lead to cardiac hypertrophy as an adaptive response^28^; hence, inhibition of actin–myosin attachment is particularly relevant to counter such hypertrophy in HCM. Reductions in LVMI and maximal LV wall thickness assessed by CMR imaging correlated with improvements in cardiac biomarkers of LV wall stress, LV outflow tract gradients, NYHA functional class, and Kansas City Cardiomyopathy Questionnaire Clinical Summary Score,^13^^,^^17^ which further supports the effect of mavacamten in improving cardiac structure, LV filling pressure, clinical symptoms, and health status. In addition to structural measures mentioned previously, improvements in functional parameters such as myocardial contraction fraction, LV stroke volume, and myocardial contraction fraction (a ratio of LV stroke volume to LV myocardial volume and an indicator of myocardial shortening predictive of cardiac adverse events)^29^ were also seen with mavacamten at week 30. Taken together, the favorable changes in cardiac output and function with mavacamten treatment were in line with the improvement in exercise capacity shown in EXPLORER-HCM. To date, the improvements in cardiac morphology and LV diastolic function seen with mavacamten have yet to be reported for other contemporary pharmacologic agents for symptomatic obstructive HCM, including disopyramide, beta-blockers, and nondihydropyridine calcium-channel blockers.^30^^,^^31^ Although successful septal reduction therapies could lead to improvements in LV wall thickness, left atrial volumes, and NT-proBNP levels,^32^^,^^33^ such invasive procedures come with inherent surgical risks. Conversely, mavacamten appears to address the underlying pathophysiology of HCM, indicated by changes in the CMR parameters presented herein, without concern of invasiveness and the complications associated with septal reduction therapies.

### Study limitations

Study limitations include the relatively small sample size and short duration of follow-up; further validation in future studies with a larger population and longer duration of follow-up is required, particularly when considering that myocardial fibrosis is a progressive process. In addition, data on the characterization of myocardial fibrosis, in terms of site and morphology, were lacking. The exploratory nature inherent with a subgroup analysis is another limitation, because this study included a subset of primarily Chinese patients with gadolinium-enhanced CMR data available from the EXPLORER-CN study. Thus, the findings may not be generalizable to a broader population. Nonetheless, baseline characteristics of these patients were consistent with the overall population in EXPLORER-CN.^17^

## Conclusions

At week 30, improvements in CMR measures related to cardiac structure, function, and volume were observed with mavacamten vs placebo. Global mass of LGE 6SD was decreased in the mavacamten group, indicative of reduced myocardial fibrosis, although this requires further validation. These results indicate favorable cardiac remodeling with mavacamten in Chinese patients with obstructive HCM, despite a short treatment period. Future studies will provide further insights into the relationship between clinical efficacy of mavacamten and changes in cardiac remodeling.

### Data Availability

Bristol Myers Squibb’s policy on data sharing may be found at https://www.bms.com/researchers-and-partners/independent-research/data-sharing-request-process.html.

## Funding Support and Author Disclosures

This work was supported by Shanghai LianBio Development Co, Ltd and Bristol Myers Squibb. The funders were also involved in study design and data collection, analysis, and interpretation, as well as review and feedback on the manuscript. Dr Fu was an employee of Shanghai LianBio Development Co, Ltd, Shanghai, China, when the study was conducted. Dr Sun is an employee of Bristol Myers Squibb. All other authors have reported that they have no relationships relevant to the contents of this paper to disclose.
