# Supplementary material for: Effects of Mavacamten on Cardiac Magnetic Resonance Features in Chinese Patients With Obstructive Hypertrophic Cardiomyopathy
Source: JACC Asia. 2025 Jul 8;5(8):1064–74. doi: 10.1016/j.jacasi.2025.05.015 (PMC12426844; doi:10.1016/j.jacasi.2025.05.015)

# Supplemental Material

# Contents

| **SUPPLEMENTAL TABLE 1** Study Eligibility Criteria | **1** |
| --- | --- |
| **SUPPLEMENTAL TABLE 2** Median (IQR) CFB in CMR Parameters at Week 30 | **11** |
| **SUPPLEMENTAL FIGURE 1** Scatter plots of correlations between change in LVM and change in global mass of LGE 6SD | **13** |
| **SUPPLEMENTAL FIGURE 2** Box Plots of Relationship Between Changes in NYHA Class From Baseline to Week 30 and CMR Parameters | **14** |

# SUPPLEMENTAL TABLE 1 Study Eligibility Criteria

| **Inclusion Criteria** |
| --- |
| Participants who met the following criteria were included in this study: |
| 1. At least 18 years old at screening. |
| 1. Body weight was greater than 45 kg at screening. |
| 1. Had adequate acoustic windows to enable accurate transthoracic echocardiographies (TTEs) (refer to echocardiography related manual). |
| 1. Diagnosed with obstructive hypertrophic cardiomyopathy (oHCM) consistent with current American College of Cardiology Foundation/American Heart Association, European Society of Cardiology, and Chinese Society of Cardiology guidelines, ie, satisfy criteria below (criteria to be documented by the echocardiography core laboratory): |
| 1. Had unexplained left ventricular (LV) hypertrophy with nondilated ventricular chambers in the absence of other cardiac (eg, hypertension, aortic stenosis) or systemic disease and with maximal LV wall thickness ≥15 mm (or ≥13 mm with positive family history of hypertrophic cardiomyopathy), as determined by core laboratory interpretation, and |
| 1. Had left ventricular outflow tract (LVOT) peak gradient ≥50 mm Hg during screening as assessed by echocardiography at rest or after Valsalva maneuver (confirmed by echocardiography core laboratory interpretation). |
| 1. Had documented LV ejection fraction (LVEF) ≥55% by echocardiography core laboratory read of screening TTE at rest. |
| 1. Had a valid measurement of Valsalva LVOT peak gradient at screening as determined by echocardiography core laboratory. |
| 1. Had New York Heart Association (NYHA) class II or III symptoms at screening. |
| 1. Had documented oxygen saturation at rest ≥90% at screening. |
| 1. Female participants should not be pregnant or lactating and, if sexually active at enrollment, had agreed upon using 1 of the following acceptable birth control methods from the screening visit through 5 months after the last dose of study treatment. |
| 1. Estrogen- and progestogen-containing hormonal contraception associated with inhibition of ovulation or progestogen-only hormonal contraception associated with inhibition of ovulation by oral, implantable, or injectable route of administration. |
| 1. Intrauterine device. |
| 1. Intrauterine hormone-releasing system. |
| 1. Bilateral tubal occlusion. |
| 1. Female: surgically sterile for 6 months or postmenopausal for 1 year. Permanent sterilization included hysterectomy, bilateral oophorectomy, bilateral salpingectomy, and/or documented bilateral tubal occlusion at least 6 months prior to screening. Females were considered postmenopausal if they have had amenorrhea for ≥1 year after cessation of all exogenous hormonal treatments, and follicle-stimulating hormone levels were in the postmenopausal range. |
| 1. Male partners of female participants must also agree to use a contraceptive (eg, barrier, condom, or vasectomy) from screening through 5 months after the last dose of study drug. |
| 1. Study participants were able to understand and comply with the study procedures, understand the risks involved in the study, and provided written informed consent according to national, local, and institutional guidelines before the first study-specific procedure. |
| **Exclusion Criteria** |
| A participant who met any of the following exclusion criteria was excluded from this study: |
| 1. Participated in a clinical trial in which the participant received any investigational drug (or was currently using an investigational device) within 30 days prior to screening, or at least 5 times the respective elimination half-life (if known), whichever was longer. |
| 1. Known infiltrative or storage disorder causing cardiac hypertrophy that mimics oHCM, such as Fabry disease, amyloidosis, or Noonan syndrome with LV hypertrophy. |
| 1. Had a history of syncope within 6 months prior to screening or sustained ventricular tachyarrhythmia with exercise within 6 months prior to screening. |
| 1. Had a history of resuscitated sudden cardiac arrest (at any time) or known history of appropriate implantable cardioverter‑defibrillator (ICD) discharge for life-threatening ventricular arrhythmia within 6 months prior to screening. |
| 1. Had paroxysmal, intermittent atrial fibrillation with atrial fibrillation present per the investigator’s evaluation of the participant’s electrocardiogram (ECG) at the time of screening. |
| 1. Had persistent or permanent atrial fibrillation not on anticoagulation for at least 4 weeks prior to screening and/or not adequately rate-controlled within 6 months prior to screening (note: participants with persistent or permanent atrial fibrillation who were anticoagulated and adequately rate-controlled were allowed). |
| 1. Previously participated in a clinical study with mavacamten. |
| 1. Hypersensitivity to any of the components of the mavacamten formulation. |
| 1. Current treatment (within 14 days prior to screening) or planned treatment during the study with disopyramide, cibenzoline, or ranolazine. |
| 1. Current treatment (within 14 days prior to screening) or planned treatment during the double-blinded treatment with a combination of beta-blockers and verapamil or a combination of beta-blockers and diltiazem. |
| 1. For individuals on beta-blockers, verapamil, or diltiazem, any dose adjustment of that medication within14 days prior to screening or any anticipated change in treatment regimen using these medications during the double-blind treatment. |
| 1. Had been successfully treated with invasive septal reduction (surgical myectomy or percutaneous alcohol septal ablation [ASA]) within 6 months prior to screening or planned to have either of these treatments during the study (note: individuals with an unsuccessful myectomy or percutaneous ASA procedure performed >6 months prior to screening were enrolled if study eligibility criteria for LVOT gradient criteria were met). |
| 1. ICD placement within 2 months prior to screening or planned ICD placement during the study. |
| 1. Had QTcF >500 msec when QRS interval <120 msec or QTcF >520 msec when QRS ≥120 msec or any other ECG abnormality considered by the investigator to pose a risk to participant safety (eg, second-degree atrioventricular block type II). |
| 1. Had documented obstructive coronary artery disease (>70% stenosis in 1 or more epicardial coronary arteries) or history of myocardial infarction. |
| 1. Had known moderate or severe (as per investigator’s judgment) aortic valve stenosis, constrictive pericarditis, or clinically significant congenital heart disease at screening. |
| 1. Had any acute or serious comorbid condition (eg, major infection or hematologic, renal, metabolic, gastrointestinal, or endocrine dysfunction) that, in the judgment of the investigator, could lead to premature termination of study participation or interfere with the measurement or interpretation of the efficacy and safety assessments in the study. |
| 1. History of malignant disease within 10 years of screening: |
| 1. Participants who had been successfully treated for nonmetastatic cutaneous squamous cell or basal cell carcinoma or had been adequately treated for cervical carcinoma in situ or breast ductal carcinoma in situ were included in the study. |
| 1. Participants with other malignancies who were cancer free for more than 10 years before screening were included in the study. |
| 1. Had safety laboratory parameters (chemistry, hematology, coagulation, and urinalysis) outside normal limits (according to the local laboratory reference range) at screening as assessed by the local laboratory; however, a participant with safety laboratory parameters outside normal limits might be included if he or she met all of the following criteria: |
| 1. The safety laboratory parameter outside normal limits was considered by the investigator to be clinically not significant. |
| 1. If there was an alanine aminotransferase or aspartate aminotransferase result, the value must be <3× the upper limit of the laboratory reference range. |
| 1. The body size–adjusted estimated glomerular filtration rate was ≥30 mL/min/1.73 m^2^. |
| 1. Had a positive serologic test at screening for infection with human immunodeficiency virus, hepatitis C virus, or hepatitis B virus surface antigen. |
| 1. Known uncured COVID-19 (coronavirus disease 2019) infection or with severe complication before screening. |
| 1. Had a history or evidence of any other clinically significant disorder, condition, or disease that, in the opinion of the investigator, would pose a risk to participant safety or interfere with the study evaluation, procedures, or completion. |
| 1. Was currently taking, or had taken within 14 days prior to screening, a prohibited medication, such as a cytochrome CYP2C19 inhibitor (eg, omeprazole or esomeprazole), a strong CYP3A4 inhibitor, or St. John’s Wort. Alternatives, such as pantoprazole were allowed and could be discussed with the medical monitor. |
| 1. Prior treatment with cardio toxic agents such as doxorubicin or similar. |
| 1. Unable to comply with the study requirements, including the number of required visits to the clinical site. |
| 1. Was a first-degree relative of personnel directly affiliated with the study at the clinical study site, any study vendor, or the study sponsor. |
| 1. Was currently taking, or had taken within 14 days prior to screening, biotin supplements (multivitamins that contain <1000 mg biotin were allowed during the study, but should be stopped 24 hours prior to each study visit). |
| 1. Identified as alcohol addicts. |
| **Cardiac magnetic resonance (CMR) exclusion criteria:** |
| A participant was to be excluded from the CMR assessments if he or she has any of the following: |
| 1. An ICD or pacemaker, or another contraindication for CMR or conditions not suitable for CMR in the judgment of the investigator. |
| 1. Atrial fibrillation at the time of screening (participant who was in atrial fibrillation at the time of imaging was asked to return later within the screening period, and if the participant was still in atrial fibrillation, the participant was disqualified from the CMR assessments). |
| 1. Allergy or contraindication to contrast medium. |
| **Removal of participants from therapy or assessment** |
| Study drug was permanently discontinued if any of the following criteria were met: |
| The following reasons would lead to permanent treatment discontinuation or withdrawal from study: |
| 1. If all the criteria are met for possible drug-induced liver injury. |
| 1. Pregnancy. |
| 1. LVEF ≤30% as determined by site laboratory. |
| 1. New or worsening heart failure associated with systolic dysfunction. |
| 1. Any breaking of the study blind requested by the investigator. |
| 1. Continued administration of study drug is considered by the investigator to be detrimental to the participant’s safety or well-being. |
| 1. The participant requests to discontinue study drug. |
| 1. The sponsor requests that the participant permanently discontinues study drug. |
| If a participant permanently discontinues treatment prior to week 30, the participants were asked to undergo an early termination visit as soon as possible after stopping study drug and were encouraged to participate in posttreatment visits (phone visit and the onsite visits) and the week 30 visit. |

# SUPPLEMENTAL TABLE 2 Median (IQR) CFB in CMR Parameters at Week 30

| **Median change (IQR) from baseline at week 30** | **Mavacamten (n = 39)** | **Placebo  (n = 19)** |
| --- | --- | --- |
| LVMI, g/m^2^ | -20.1 (-35.2, -11.8) | 4.2 (-1.4, 12.8) |
| LV mass, g | -37.8 (-67.3, -20.1) | 6.9 (-1.5, 19.0) |
| Maximal LV wall thickness, mm | -2.7 (-4.3, -1.1) | 0.4 (-0.2, 1.4) |
| Maximum LAVI, mL/m^2^ | -13.5 (-32.7, -4.6) | -0.7 (-6.7, 12.1) |
| Minimum LAVI, mL/m^2^ | -9.6 (-17.3, -1.2) | 1.0 (-5.6, 5.8) |
| LVEF, % | -3.3 (-11.2, 2.1) | -1.0 (-4.6, 1.4) |
| Myocardial contraction fraction, % | 11.0 (-3.6, 19.5) | -4.8 (-12.6, 2.6) |
| Contractile fraction, % | 10.5 (-3.5, 18.6) | -4.5 (-12.0, 2.5) |
| LVEDV, mL | -18.9 (-28.5, -3.3) | 2.2 (-8.2, 10.1) |
| LVEDV index, mL/m^2^ | -9.9 (-14.5, -1.5) | 1.5 (-1.5, 5.6) |
| LVESV, mL | 0.03 (-4.6, 10.4) | 3.1 (-2.7, 6.0) |
| LVESV index, mL/m^2^ | -0.3 (-3.1, 6.8) | 1.7 (-1.4, 4.2) |
| LVSV, mL | -17.9 (-27.4, -6.1) | 0.8 (-13.9, 8.2) |
| LVSV index, mL/m^2^ | -10.2 (-14.9, -2.5) | 0.4 (-6.6, 5.6) |
| CO, mL/min | -640.3 (-2065.6, -339.4) | 160.0 (-1708.5, 1153.7) |
| COI, mL/min/m^2^ | -366.6 (-1153.7, -139.7) | 58.2 (-796.4, 613.5) |
| Global mass of LGE 6SD, g^†^ | -0.8 (-4.5, 0.0) | 0.6 (0.0, 3.2) |
| 5-min global ECVF, %^‡^ | 2.2 (0.1, 3.6) | 1.1 (-2.1, 2.2) |
| 10-min global ECVF, %^§^ | 1.2 (-0.9, 3.0) | 0.4 (-1.6, 2.4) |
| 25-min global ECVF, %^\|\|^ | 1.1 (-0.7, 2.3) | -0.2 (-1.1, 1.9) |

^†^36 patients on mavacamten and 15 on placebo at baseline, and 32 and 14 patients at week 30, respectively. ^‡^35 patients on mavacamten and 17 on placebo at baseline, and 34 and 16 patients at week 30, respectively. ^§^36 patients on mavacamten and 18 on placebo at baseline, and 34 and 17 patients at week 30, respectively. ^||^38 patients on mavacamten and 19 on placebo at baseline, and 37 and 16 patients at week 30, respectively.

CFB = changes from baseline; CMR = cardiac magnetic resonance; CO = cardiac output; COI = cardiac output index; ECVF = extracellular volume fraction; IQR = interquartile range; LAVI = left atrial volume index; LGE 6SD = late gadolinium enhancement by 6 standard deviations; LV = left ventricular; LVEDV = LV end-diastolic volume; LVEF = LV ejection fraction; LVESV = LV end-systolic volume; LVMI = LV mass index; LVSV = LV stroke volume.

# SUPPLEMENTAL FIGURE 1 Scatter plots of correlations between change in LVM and change in global mass of LGE 6SD

Change in global mass of LGE 6SD appeared to be independent of change in LVM. Shaded area represents 95% CI of the respective regression line. Correlation shows Pearson correlation coefficient. Number of patients with available data: Mavacamten, n = 32, placebo, n = 14

CI = confidence interval; LVM = LV mass; LGE 6SD = late gadolinium enhancement by 6 standard deviations


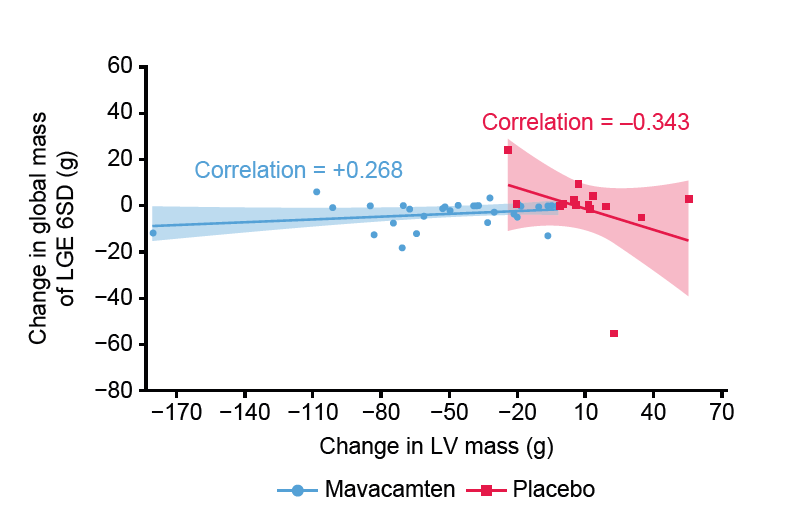


# SUPPLEMENTAL FIGURE 2 Box Plots of Relationship Between Changes in NYHA Class From Baseline to Week 30 and CMR Parameters

Improvement in NYHA class appeared to correlate with improvement in LVMI (A), global maximal LV wall thickness (B), LVEDVI (C), LAVI maximum (D), and global mass of LGE 6SD (E). CFB = change from baseline; CMR = cardiac magnetic resonance; LAVI = left atrial volume index; LGE 6SD = late gadolinium enhancement by 6 standard deviations; LV = left ventricular; LVEDVI = left ventricular end-diastolic volume index; LVMI = left ventricular mass index; NYHA = New York Heart Association.


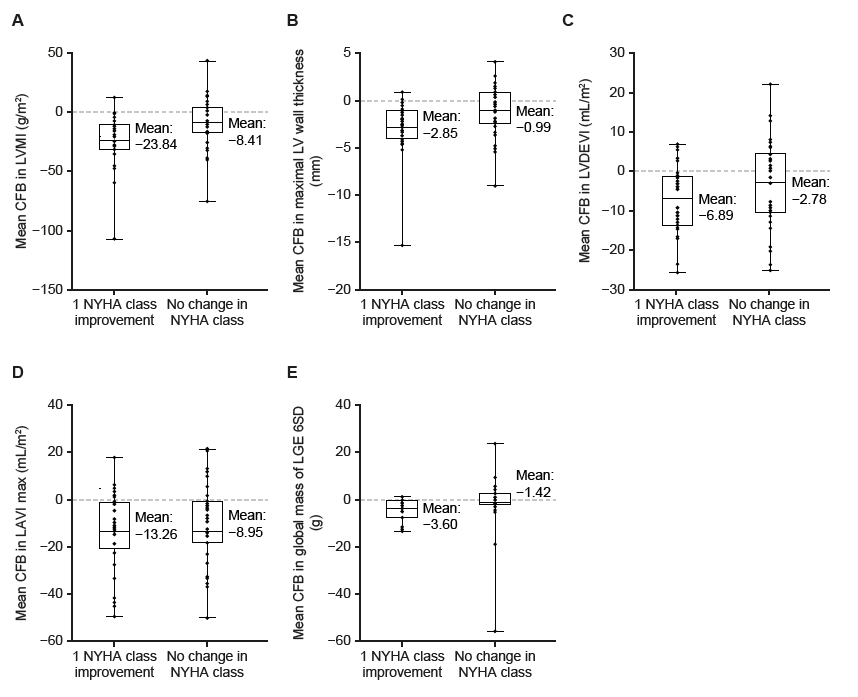

Supplement: Supplemental Tables 1 and 2 and Supplemental Figures 1 and 2 [file mmc1.docx]
